# Physical Hazards in Employment and Pregnancy Outcome

**DOI:** 10.4103/0970-0218.51224

**Published:** 2009-04

**Authors:** Bratati Banerjee

**Affiliations:** Department of Community Medicine, Maulana Azad Medical College, Bahadur Shah Zafar Marg, New Delhi - 110 002, India

## Introduction

The past century has seen an enormous improvement in the political, social, and economic status of women, of which employment is an integral part.

Women workers require special protection while pregnant because of the following reasons:(1)

1. The developing embryo may be more susceptible to noxious agents than the exposed mother.
2. Females may be less suited for some work tasks than males.
3. Pregnancy may decrease the capacity to cope with many work factors.
4. Women tend to feed themselves less substantially than men.

## The Physiology of Work During Pregnancy

Physical exertion has often been suggested as a risk factor for adverse pregnancy outcomes,(2–21) which may occur due to various factors.(4,22–24)

1. Work involves muscle action, causing an increase of sympathetic vasomotor activity in working muscles proportional to the severity of work. Hence, when cardiac output increases rapidly during muscular activity, most blood goes to the working muscles and proportionately less arrives in the other viscera, which during pregnancy, includes the placental bed.
2. With hard work there is an increased release of catecholamines with resultant arteriolar constriction. With chronic hard work, lactate accumulates making any unloading of lactate from the fetus more difficult.
3. Sweating reduces plasma volume, which is an important determinant of uteroplacental blood flow. Circulatory blood flow in the uterus and placenta decreases in the standing position.
4. Pronounced physical exercise may lead to hormonal disturbances, hypothermia, and nutritional deficits.
5. Shift work and irregular work schedules could lead to a change in the circadian rhythm, which affects regulation of the ovulatory cycle with resultant menstrual irregularities. Whether such shifts also influence pregnancy outcome is unknown.
6. Heavy lifting increases intra-abdominal pressure and this may provoke uterine contractions.
7. Heat stress causes dehydration resulting in the release of the anti-diuretic hormone and oxytocin, which stimulates the uterus to contract.

Due to the combined effects of vasoconstriction, myometrial contraction, reduced plasma volume, and diversion of blood flow away from the placental bed, there is diminution of uteroplacental blood flow and resultant fetal hypoxia. This, along with possible hormonal imbalance, may have deleterious effects on the fetus.

## Reproductive Risk Factors

### Heavy lifting

Exposure to heavy lifting may be classified as follows:(3)

**Table:** 

Grade	Work exposure
0	No heavy lifting at work
I	Heavy lifting (any weight), less than 10 times per week
II	Heavy lifting less than 12 kg, more than 10 times per week
III	Lifting 12 kg or more, 10 to 50 times per week
IV	Lifting 12 kg or more, more than 50 times per week

### Shift work and inconvenient working hours

These may be defined as the following:(4)

Inconvenient working hours - any work outside the period of 0645 - 1745 hours.

Irregular working hours - work outside this interval, not beginning or ending at the same time each day.

### Long working hours

Workers have been classified as full-time and part-time.(25)

Full time - those who work 35 hours or more per week

Part time - those who work less than 35 hours per week

### Time of stopping work

Heavy lifting may have a negative effect on a child's birth weight if work is continued until the 32^nd^ week of pregnancy or longer.(3)

### Work exposure

This may involve prolonged bending, stooping, twisting, or standing. Work may also be classified as sedentary or non sedentary based on the amount of physical work expended.(26,27)

### Other physical effort

Physical effort in any form that causes fatigue.(27)

### Psychosocial stress

This has been defined as job control, job demands, and work-related physical exertion.(26)

### Environmental exposure

Exposure to the extremes of temperature, noise, and vibration.(28)

## Work Fatigue Score

Several physical and ergonomic factors, studied as separate entities, have been considered as reproductive risk factors. Mamelle, *et al.* defined a fatigue scoring system by accumulating all such factors with a score of 1 allotted for the presence of each index factor as follows:(29,30)

1. Posture
2. Work on machine
3. Physical exertion
4. Mental stress
5. Environment

Another similar system included the following working conditions:(31)

1. Standing for the majority of work
2. Carrying heavy loads
3. Working on an assembly line
4. Performing a physically demanding job

## Women in Employment and Their Pregnancy

A possible influence for the motivation for employment by women is related to personal fulfillment of economic necessity - “privilege effect” or “desperation effect”. Studies conducted on women of varying employment status show multiple demographic and behavioral differences. Employed women are generally younger, more highly educated, have a higher family income, are less likely to be married, have half the number of pregnancies reported by housewives, begin prenatal care earlier, miss fewer antenatal appointments, and gain more weight during pregnancy. They smoke less, both early and late in pregnancy, but light and moderate alcohol consumption is often more.(25,32)

The hypothesis that women's increased work participation may have significant health consequences is based upon two observations. First, women do not generally shed their housekeeping and child rearing responsibilities when they enter the workforce, resulting in exhaustion. Second, pregnant working women may come in contact with an environment that may damage the fetus.(32)

## Physical Work During Pregnancy and its Hazard to the Fetus

Women in various occupations have been found to be at an increased risk of experiencing a fetal death. Of all the sectors observed, risk was highest for those employed in the textile industry.(5–21)

The significant work factors directly correlated with adverse pregnancy outcomes included: fewer household helpers, standing at work for more than 7 hours per day, working in hot environments, commuting, walking, and carrying and lifting heavy weight.(2,33–35)

A meta-analysis has shown physically demanding work to be significantly associated with pre-term birth. Other occupational exposures significantly associated included prolonged standing, shift and night work, and a high cumulative work fatigue score. No significant association was found between long work hours and pre-term birth.(36)

Standing at work and lifting heavy weight, though often not significant, have been seen to be associated with pregnancy loss and pre-term birth, especially in women whose nutrition status is compromised.(5–9,18,37–40)

Risk of pregnancy loss has been observed to be higher among women with a fixed evening work schedule in comparison with women with a fixed day schedule, and twice as high among those on a fixed night schedule. The highest abortion rate has also been seen in workers who reported irregular working hours and rotating shifts.(41–43) Effects of rotating shift work on both gestational age and birth weight have also been found to be significant.(44)

Women working more than 45 hours per week are 5 times more likely to report high stress as those working less than 35 hours per week.(42,45–47)

Environmental influence on pregnancy outcome has also been observed. Long-term whole body vibration exposures and working in extremely cold environments can contribute to disturbances of pregnancy causing abortion and stillbirth.(16,48) Occupational noise above 85-90 dB was associated with a decline in birth weight.(49,50)

Some studies, however, have not reported higher risks, and employed women sometimes have been observed to have fewer previous miscarriages or perinatal deaths when compared with housewives. Also, the higher rate of previous miscarriages among working women than non working women, observed in one study, had disappeared when adjusted for parity.(32,51,52)

The risk of employment on delivering a low birth weight infant has also been negated by many authors. No difference was observed in these aspects between economically active women compared with inactive women, or in women with stress, anxiety, depression, and physical strain due to employment, than in those without.(53–56)

## Validity of the Studies

Studies of occupational exposures and reproductive health have reported varied results. This may be due to the following reasons:(57)

- Many studies are small in terms of the number of women exposed and the number of abnormal outcomes.
- The number of potential association investigated is large and as a result some may appear 'statistically significant'.
- A large number of non-occupational factors, many still unknown, affect the pregnancy outcome.
- Reasons for traumatic events, such as fetal death or serious defect, may be influenced by the traumatic experience.

Several types of potential bias also threaten the validity of such studies.(25,58)

1. Healthy pregnant worker effect
  - Favorable social, economic, and behavioral aspects of employment, like income from employment, better health insurance coverage, better prenatal care, and a more healthy lifestyle, are expected to be associated with favorable pregnancy outcomes among working women.
  - Women with severe illness or mental incapacity may be selected out of the workforce leaving healthier working women with the possibility of better reproductive outcomes.
  - Women with a history of adverse reproductive outcomes avoid employment for fear of repeated pregnancy problems.
2. Unhealthy pregnant worker effect
  - Women who have healthy infants may stop work while their children are young, whereas women who fail to produce a surviving infant may be more likely to continue working.

These studies are often affected by recall bias and 75% of recorded abortions are recalled. Recall failure is influenced by factors such as the time elapsed since the event, total number of births or spontaneous abortions, the woman's age at the time of pregnancy, times in a woman's life when abortions occur, and the gestational age of the fetus at the time of abortion. Medical treatment received and hospital admission might emphasize the event and facilitate recall. Social class and education are other factors that may affect the completeness of the recall. Also, there are chances of misrepresentation of exposure data obtained by the self-administered questionnaire.(3,59,60)

## Recommendations

During work, some inevitable adverse conditions may arise. Various authors have suggested measures to make these conditions more bearable and less harmful for the pregnant worker and their children.(61–65) Accordingly, the following recommendations may be made:

1. Food - Reproductive risks are more pronounced in malnourished women, therefore, supplementary nutrition may be given to all pregnant working women to provide a part of their daily calorie and protein requirement.
2. Rest - Rest rooms should be provided near the workplace where pregnant women can rest when fatigued. Pregnant women should also be allowed 10 minutes rest after every 1 hour of work. Rush hours can be avoided by coming after the beginning of the shift and leaving after the shift is over.
3. Leave - Leave rules should be brought to the knowledge of all workers and they should be advised to avail such leaves.
4. Antenatal care - Antenatal clinics may be arranged within factory premises or several smaller establishments together may hold an antenatal clinic regularly. Women should be allowed paid time off to attend these clinics and travel assistance should be provided for referred cases. Investigations and medications should be provided by the employer. One health worker may be engaged to regularly visit the pregnant women.
5. Intra natal and post natal care - In India, these are generally rendered by ESI hospitals. Vehicles should be provided to attend the hospital during an emergency. For others, traveling allowance should be sanctioned.
6. Family planning - Such clinics may be held within the premises for distributing temporary contraceptive devices.
7. Risk factors - Measures should be adopted to avoid exposure of pregnant women to work conditions considered to be reproductive risk factors. All female workers should be trained for working in all departments where women are usually posted. Pregnant women should be assigned work with the least physical strain, and allotted work in fixed day shifts only, throughout their pregnancy. Sitting arrangements, fans, cool drinking water, and ear muffs should be provided.
8. Orientation programs - Such programs should be held regularly for all women workers for the purpose of generating awareness and motivating them to utilize available facilities. The employer and managerial and supervisory staff should have adequate knowledge of the issue and they should see that the facilities are being availed properly, without misuse by ineligible workers.
9. Awareness of community - Mass media can be used to improve the knowledge of all employers and employees regarding reproductive health.
10. Research -Reproductive research should be promoted. Governmental and non governmental research organizations should be given more assistance for work in this sphere.
